# No difference in small bowel microbiota between patients with irritable bowel syndrome and healthy controls

**DOI:** 10.1038/srep08508

**Published:** 2015-02-17

**Authors:** Aldona Dlugosz, Björn Winckler, Elin Lundin, Katherina Zakikhany, Gunnar Sandström, Weimin Ye, Lars Engstrand, Greger Lindberg

**Affiliations:** 1 Karolinska Institutet, Department of Medicine and Center for Digestive Diseases, Karolinska University Hospital Huddinge, Stockholm, Sweden; 2 Karolinska Institutet, Department of Medical Epidemiology and Biostatistics, Stockholm, Sweden; 3 Stockholm University, Department of Biochemistry and Biophysics, Stockholm, Sweden; 4 Public Health Agency of Sweden, Unit for Laboratory Surveillance of Vaccine Preventable Diseases, Solna, Sweden; 5 Karolinska Institutet, Department of Laboratory Medicine, Stockholm, Sweden; 6 Karolinska Institutet, Department of Microbiology, Tumor & Cell Biology and Science for Life Laboratory, Stockholm, Sweden

## Abstract

Several studies have indicated that colonic microbiota may exhibit important differences between patients with irritable bowel syndrome (IBS) and healthy controls. Less is known about the microbiota of the small bowel. We used massive parallel sequencing to explore the composition of small bowel mucosa-associated microbiota in patients with IBS and healthy controls. We analysed capsule biopsies from the jejunum of 35 patients (26 females) with IBS aged 18-(36)-57 years and 16 healthy volunteers (11 females) aged 20-(32)-48 years. Sequences were analysed based on taxonomic classification. The phyla with the highest total abundance across all samples were: Firmicutes (43%), Proteobacteria (23%), Bacteroidetes (15%), Actinobacteria (9.3%) and Fusobacteria (7.0%). The most abundant genera were: *Streptococcus* (19%), *Veillonella* (13%), *Prevotella* (12%), *Rothia* (6.4%), *Haemophilus* (5.7%), *Actinobacillus* (5.5%), *Escherichia* (4.6%) and *Fusobacterium* (4.3%). We found no difference among major phyla or genera between patients with IBS and controls. We identified a cluster of samples in the small bowel microbiota dominated by *Prevotella*, which may represent a common enterotype of the upper small intestine*.* The remaining samples formed a gradient, dominated by *Streptococcus* at one end and *Escherichia* at the other*.*

The human gastrointestinal microbiota consists of about 100 trillion microbial cells that outnumber our own cells by a factor of 10^1^. In a healthy host, bacteria colonize the alimentary tract soon after birth, and the composition of the intestinal microflora is believed to remain relatively constant throughout life^2^. The adult human intestine is home to more than 1000 species of microbes, which normally remain confined to the distal gut (colon) where the concentration of organisms is approximately 10^11^ organisms per gram of content^3^,^4^. Because of peristalsis and the antimicrobial effects of gastric acidity, the stomach and proximal small intestine contain small numbers of bacteria in healthy individuals. The bacterial counts of coliforms rarely exceed 10^3^ colony-forming units (CFU)/mL in jejunal juice^2^.

In a study based on human faecal samples that spanned several nations and continents (totally 39 individuals) Arumugam *et al*.^5^ demonstrated the existence of enterotypes in the human gut microbiome and identified three of them that varied in species and functional composition. Each of these three enterotypes are identifiable by the dominance of one of three genera: *Bacteroides* (enterotype 1), *Prevotella* (enterotype 2) and *Ruminococcus* (enterotype 3). The relationships between microbiota and different diseases and conditions have been studied especially in the colonic microbiota and some significant associations have been observed for inflammatory bowel diseases (IBD)^4^,^6^, metabolic syndrome^7^, and obesity^8^.

The small bowel microbiota has not been fully described, partly because small bowel samples are relatively difficult to obtain^9^. The microbiota of effluents from the distal small bowel was found to vary with the intake of carbohydrates in a single patient with ileostomy^10^. Another study found a substantial difference between morning and afternoon samples of the ileal effluent in one subject with ileostomy and the variation in the microbiota profile during the day was larger than that seen in repeat samples obtained at the same time of the day over 9–28 days in 4 subjects^11^. The suspicion that host-microbe interaction may underlie observed immune activation in IBS makes the mucosa-associated microbiota more interesting as target for research than luminal microbiota^12^.

IBS is a common gastrointestinal disorder characterized by abdominal pain or discomfort and altered bowel function^13^. The potential differences of the intestinal microbiota between IBS patients and healthy controls have mostly been studied using stool samples, as this is the most accessible source of the GI microbiota^14^,^15^. In a detailed faecal microbiota analysis of a well-characterized cohort of IBS patients Jeffery *et al*.^16^ identified several clear associations with clinical data and a distinct subset of IBS patients with alterations in their microbiota that did not correspond to IBS subtypes, as defined by the Rome-II criteria^13^.

Kerckhoffs et al.^17^ found decreased *Bifidobacteria* levels in both faecal and duodenal brush samples of IBS patients compared to healthy subjects. Small intestinal bacterial overgrowth (SIBO) was proposed to be common in IBS^18^. Bacterial overgrowth is a condition caused by an abnormal number of bacteria in the small intestine, exceeding 10^5^ organisms/ml (5 log colony-forming units (CFU)/ml) owing to different predisposing conditions, such as impaired motility or failure of the gastric-acid barrier^19^,^20^. The direct aspiration and culture of jejunal fluid, with results expressed as CFU/mL of jejunal fluid has been regarded by many investigators as the gold standard for the diagnosis of SIBO, but molecular techniques suggest that as much as 80% of the normal flora is not identified by culture-based methods^2^. Profiling the microbiome using methods based on the 16S ribosomal RNA gene is less biased than cultivation based approaches. In particular, pyrosequencing using parallel bar-coded sequence tags enables deep sequencing of multiple samples and provides high taxonomic resolution^21^. The power of pyrotag sequencing for exploration of the human microbiome has been shown for different body sites^21^. As faecal samples are not representative of the entire intestine, the aim of the present study was to deeply explore the composition of small bowel mucosa-associated microbiota using 454-barcoded pyrosequencing in patients with IBS compared to healthy controls.

## Results

The amplicon reads were preprocessed as described in the “Bioinformatics pipeline” section, resulting in a data set consisting of 51 samples and 350 OTUs. The median depth of samples was 3771 and the minimum depth was 628. More than 99% of all reads could be taxonomically classified to phylum rank and 90% up to genus rank.

**Figure 1:**
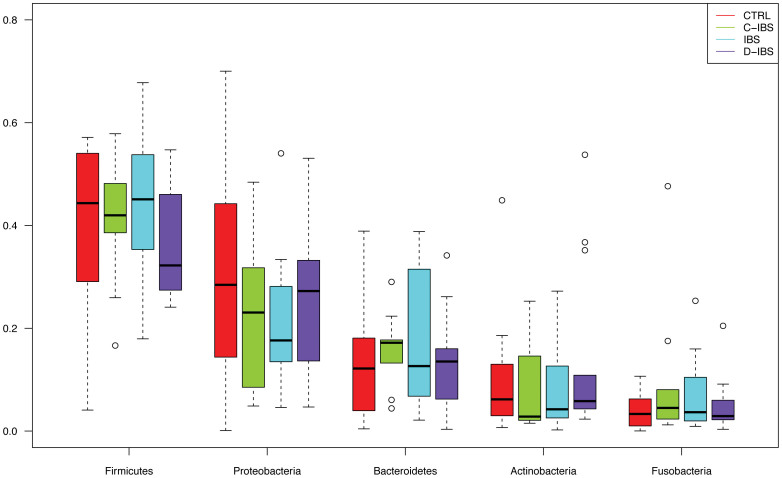
Relative abundance of the five most common phyla among IBS patients and controls.

**Figure 2:**
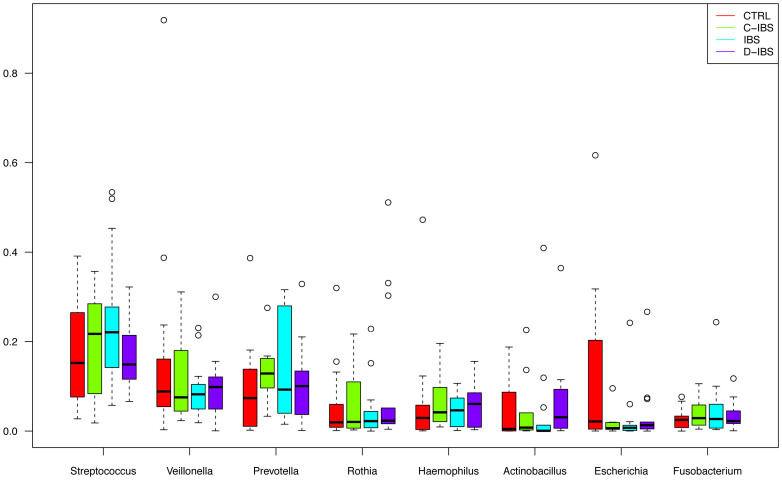
Relative abundance of the eight most common genera among IBS patients and controls.

The phyla with the highest total abundance across all samples were: Firmicutes (43%), Proteobacteria (23%), Bacteroidetes (15%), Actinobacteria (9.3%) and Fusobacteria (7.0%) (Figure 1). These phyla were present in all samples. The most abundant genera were: *Streptococcus* (19%), *Veillonella* (13%), *Prevotella* (12%), *Rothia* (6.4%), *Haemophilus* (5.7%), *Actinobacillus* (5.5%), *Escherichia* (4.6%) and *Fusobacterium* (4.3%) (Figure 2). These genera were present in more than 98% of all samples, except *Actinobacillus*, which was present in 82%.

In order to test if there were any differences in terms of bacterial diversity within samples (*α*-diversity) we looked for associations between the disease phenotype (control, IBS, C-IBS, D-IBS) and the number of OTUs, as well as the Chao1 and Shannon diversity. It is well known that these measures are highly dependent on sequencing depth^22^, so we first randomly sub-sampled all samples to have even depth before evaluating the above measures of diversity. We found no statistically significant difference between disease phenotypes in terms of *α*-diversity.

We used Metastats^23^ in order to detect bacteria that were differentially expressed across controls and patients, as well as across the different disease phenotypes. Although we found no statistically significant difference between IBS and controls, we did find some OTUs that exhibited a trend towards differential expression (Table 1). None of these differences was significant after adjusting for multiple testing. Since these OTUs are abundant we can rule out the possibility that observed differences were due to insufficient sampling depth but more samples would be needed to determine whether differences are real or due to chance.

**Figure 3:**
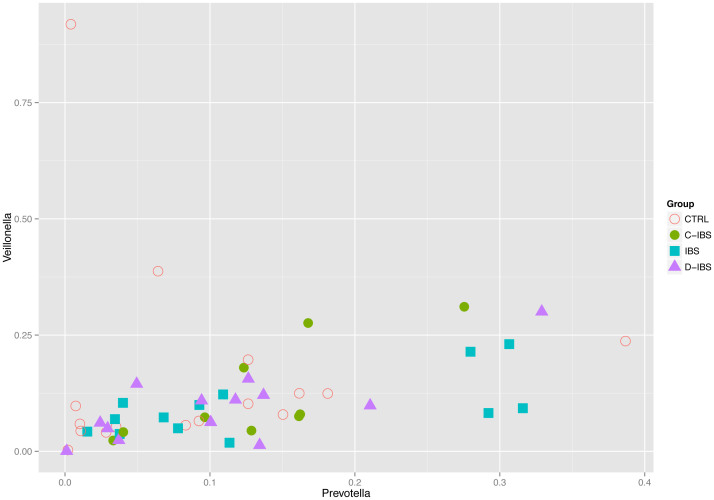
Correlation between the relative abundance of *Prevotella* and *Veillonella* (Spearman rho = 0.55 *ρ* ≈ 0.55, *p* ≈ 4 × 10^−5^).

**Figure 4:**
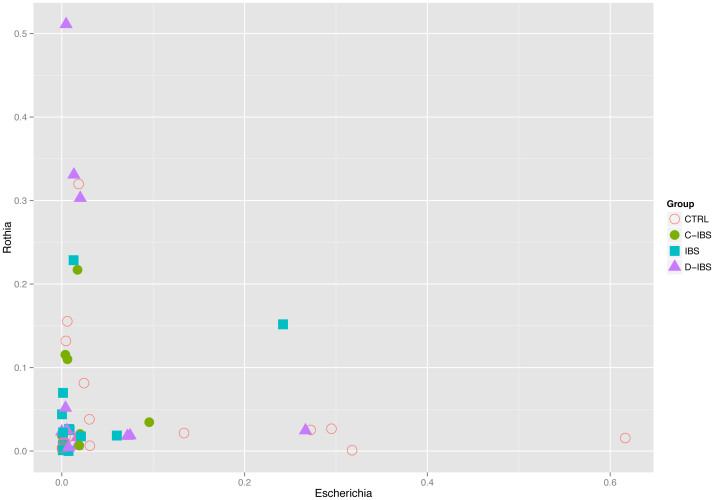
The relative abundance of *Escherichia* and *Rothia* exhibit a mutually exclusive relationship.

**Figure 5:**
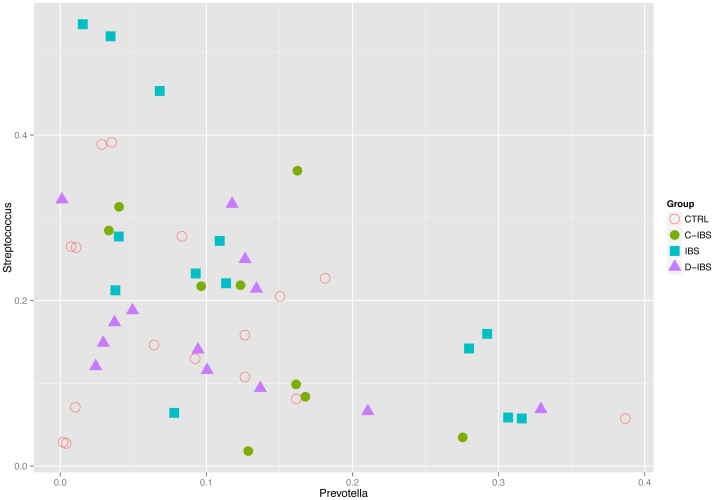
The relative abundance of *Prevotella* and *Streptococcus* exhibit an inverse relationship in samples with high total abundance of these two genera.

**Figure 6:**
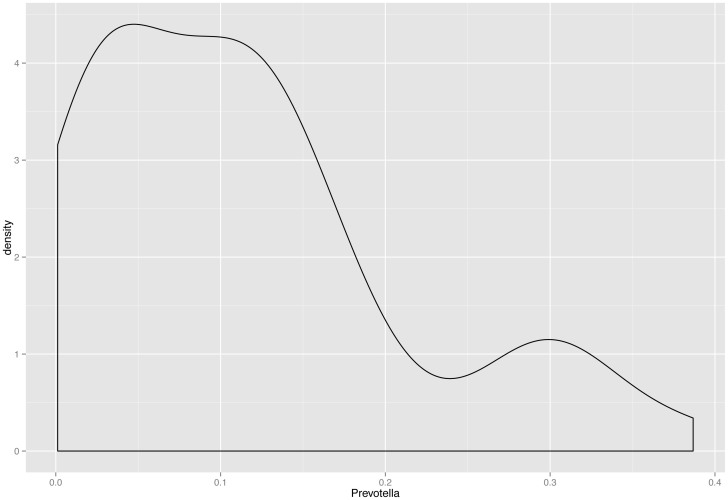
The distribution of *Prevotella* abundance is bimodal, indicating that samples may be naturally subdivided into two distinct subgroups according to whether they have low or high *Prevotella* abundance.

We found that the abundance of certain genera exhibited patterns of co-dependence. In particular: (1) *Prevotella* and *Veillonella* were correlated (Spearman *ρ* ≈ 0.55, *p* ≈ 4 × 10^−5^) (Figure 3), (2) *Escherichia* and *Rothia* seemed to be mutually exclusive in the sense that more than 5% of one typically implied less than 4% of the other (Figure 4), and (3) samples in which the total abundance of *Prevotella* and *Streptococcus* was high exhibited an inverse relationship between these genera (Figure 5). The distribution of *Prevotella* abundance was bimodal (Figure 6), indicating that samples may be naturally subdivided into two distinct subgroups according to whether they have low or high *Prevotella* abundance.

A cluster analysis was performed in order to check for evidence of a *Prevotella* “enterotype” as described by Arumugam *et al*.^5^. We considered Bray-Curtis distance (BC) and Jensen-Shannon divergence (JSD) on the relative abundance aggregated to genus level, as well as weighted and unweighted UniFrac (after subsampling all samples to have even depth). Coordinates for each dissimilarity matrix were estimated using classical multidimensional scaling (MDS/PCoA) and these coordinates were clustered using partitioning around medoids (PAM). We found one robust cluster consisting of roughly 20%–27% of all samples, including all the samples that were enriched for *Prevotella* (in line with the bimodal distribution of *Prevotella* abundance mentioned above). By “robust” we mean that this cluster appeared for all dissimilarities apart from unweighted UniFrac (Supplementary Figure 1) and for a range of choices of the number of clusters (which is a parameter to the PAM algorithm). We also tested the clustering strength using prediction strength (PS), silhouette index (SI) and Calinski-Harabasz coefficient (CH)^24^,^25^,^26^. While PS and SI were never strong (maxima around 0.6 and 0.3, respectively) we always found the robust cluster for the value at which CH (or PS, or SI) were maximal.

**Figure 7:**
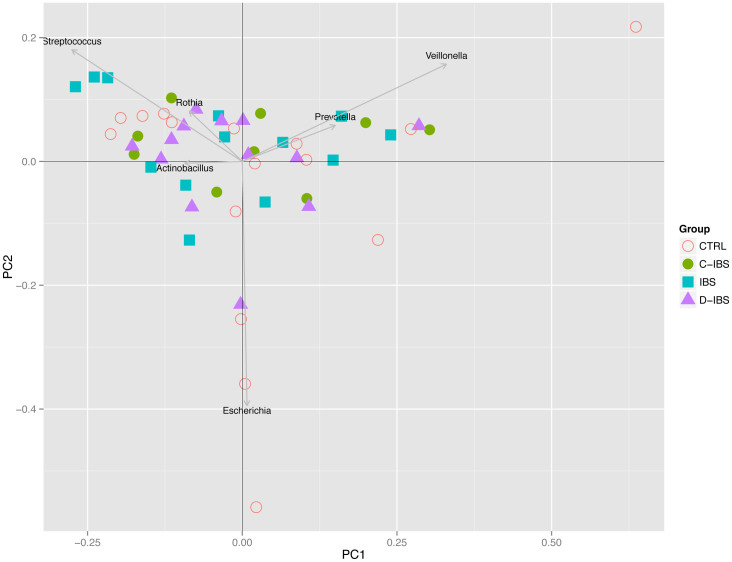
A principal component analysis of all samples. The first two principal components were roughly organized along three directions determined by enrichment for *Prevotella*, *Streptococcus* or *Escherichia*.

The fact that the robust cluster appears for several dissimilarities and different choices of the number of clusters we take as evidence in favour of a *Prevotella* enterotype. However, the low clustering strength indicates that the separation from other samples is not definite. Samples outside the robust cluster seemed to be distributed more along a gradient whose extremes typically were enriched for *Streptococcus* on the one end and *Escherichia* on the other. A principal component analysis supported the above observations (Figure 7). The first two principal components were roughly organized along three directions determined by enrichment for *Prevotella*, *Streptococcus* or *Escherichia*.

We investigated the possible association between the classification into control or patient and MDS coordinates by plotting two coordinates at a time. Looking at such plots it seemed that controls had a tendency to be spread out away from patients. To confirm this visual indication, we performed logistic regressions with the first five MDS coordinates as independent variables and the classification into control or patient as the dependent variable. The statistical models for BC (Supplementary Figure 2A), JSD (Supplementary Figure 2B) and UniFrac (Supplementary Figure 2C) all indicated that MDS coordinate 2 exhibited a non-significant trend towards separation of controls from patients, *p* ≈ 0.08 (BC, UniFrac) and *p* ≈ 0.07 (JSD) after adjusting for age and gender. No evidence of association was found for weighted UniFrac.

In order to look for differences between individual IBS phenotypes we performed a multinomial logistic regression (adjusted for age and gender) with the first five MDS coordinates as independent variables and the disease phenotype as dependent variable. We did not find any indication that different IBS phenotypes could be separated in this way.

## Discussion

Our study is the first attempt at characterizing small bowel microbiota with massive parallel sequencing. We detected representatives of several genera with a predominance of Firmicutes. The prevalence of phyla in the jejunum was different from that in the stomach analysed by Andersson *et al*.^21^ using the same methodology. At the taxon level *Streptococcus spp*., *Veillonella spp.*, *Prevotella spp*., *Rothia spp*., *Haemophilus spp*., *Actinobacillus spp., Escherichia spp. and Fusobacterium spp.* were the dominating species. Our results differ from those obtained by Zilberstain *et al*.^27^ in a study based on the direct aspiration and culture of jejunal fluid, describing domination of *Veillonella spp.*, *Lactobacillus spp*., *Proteus spp*., and *Bacteroides spp*. in proximal jejunum. This difference is expected, since Zilberstain used culturing as the method of choice to characterize the microbiota and it is known that only a part of the bacterial colonizers of the gut can be cultured^28^,^29^. One previous study that used PCR-amplified 16S rDNA clone libraries to characterize the microbial diversity of a jejunum biopsy from a single healthy subject found 78% Firmicutes, 13% Proteobacteria, 3% Bacteroidetes, 1% Actinobacteria, and 3% Fusobacteria^30^. Although these abundances were somewhat different from our mean values, they all fell within the ranges observed in our material.

454-Barcoded pyrosequencing is a powerful method to explore the diversity within the human gut ecosystems but it also has some limitations. One of them is the inability to distinguish between live and dead bacteria. Using jejunum biopsies instead of jejunal fluid we analysed mucosa-associated bacteria, thus diminishing the possibility of sequencing bacteria just passing the gastrointestinal tract. The mucosa-associated bacteria are of particular interest since they, generally speaking, are more likely to have a direct effect on the host through the mucosal layer than the bacteria only passing through the intestinal tract. We cannot exclude the contamination from oral or oesophageal flora although the probability is very low. We washed the Watson capsules before opening them and our method for DNA extraction was designed to extract only mucosa-associated flora. However, we found similarities at both phylum and taxon level with previously described microbiota from the distal oesophagus^31^. Franzosa *et al*.^32^ identified a subset of abundant oral microbes that routinely survive transit to the gut, but with a minimal transcriptional activity there. Although DNA from oral species was detectable in the gut it did not form a dominant component of that community.

We found no significant difference in small bowel microbiota between IBS patients and healthy controls. Our results do not correlate with some previous studies that reported differences between IBS patients and healthy controls in the composition of faecal microbiota^14^,^33^,^34^. Moreover, our findings do not support a role for SIBO in IBS. We did not find any qualitative differences in jejunum microbiota between patients and controls although we cannot exclude quantitative differences because the 16S amplicon cannot measure the absolute number of bacteria. The relationship between SIBO and IBS is highly inconsistent among studies^35^. SIBO is often diagnosed on the basis of various techniques for carbohydrate breath testing^36^. However, in a recent study Yu *et al*.^37^ demonstrated that lactulose breath testing detects oro-caecal transit, not small intestinal bacterial overgrowth in patients with IBS.

The achieved results do not reveal pronounced and reproducible IBS-related deviations of entire phylogenetic or functional microbial groups. The lack of apparent similarities in the taxonomy of microbiota in IBS patients may partially arise from the fact that the applied molecular methods, the nature and location of IBS subjects, and the statistical power of previous studies have varied considerably^15^. It is unclear whether IBS is a disorder of the small intestine or the large intestine, or both. Our findings do not support a role for microbiota of the upper small intestine in the pathogenesis of IBS. However, we cannot rule out that changes in microbiota of the distal small bowel might influence development of IBS or IBS symptoms. Several studies have indicated that both epithelial barrier function and enteroendocrine function of the small intestine are important players in the crosstalk between intestinal microbes and the host that is believed to have an important role in the development of IBS^38^.

Regarding the degree of inter-individual variation of the gut microbiota in IBS, previous studies reported a highly significant loss of variation in IBS patients^39^ or suggested that the microbiota of IBS subjects was more heterogeneous than that of healthy controls^40^. We could not confirm such findings in our study.

Our study lends some support to the existence of a *Prevotella* enterotype in the small bowel but not the other two enterotypes described by Arumugam *et al*.^5^ This observation is in line with new data suggesting that the boundaries between the enterotypes may be fuzzier than previously suggested and the communities of gut bacteria may form a spectrum rather than falling into distinct groups^41^,^42^. The discrepancy can also be explained by different materials analysed in the two studies: faecal samples in the Arumugan study and small bowel mucosa samples in ours.

It is unclear whether or not *Bacteroides* versus *Prevotella* enterotypes exist as distinct entities or rather represent a continuum where the observed dietary associations occur at the extremes. Perhaps better described as an enterogradient between abundance of *Bacteroides*- and *Prevotella*- dominant gut microbial communities, it currently appears that these two genera do not coexist well within the gut environment. Organisms that are phylogenetically related and functionally similar tend to coexist within the same environment consistent with niche-driven community structures. Coexclusion of *Bacteroides* and *Prevotella*, taxonomically and functionally similar genera, within the gut is an exception perhaps suggesting competition within the same niche^43^. We cannot exclude that *Prevotella* enterotype reflects small bowel microbiota, as we found in the present study, and *Bacteroides* large bowel microbiota. The abundance of *Bacteroides* versus *Prevotella* may be an oversimplification of alternative states of the gut microbiota in response to diet^44^.

Our study is the first to characterize small bowel microbiota with massive parallel sequencing. We did not confirm significant differences in small bowel mucosa-associated microbiota between patients with IBS and healthy individuals although we identified candidates for potentially being differentially expressed between controls and patients. Further studies are required to verify our results.

## Methods

### Patients

All patients fulfilled Rome-II criteria for IBS^13^. A total of 35 patients (26 females) with a median age of 36 (range 18–50) were investigated. Diarrhoea-predominant IBS (D-IBS) was present in 13 patients (37%), while 9 patients (26%) had constipation-predominant IBS (C-IBS) and 13 patients (37%) had IBS that did not fulfil the criteria for D-IBS or C-IBS.

### Controls

The control group comprised 16 healthy volunteers (11 females) in whom presence of IBS and all other functional bowel disorders had been excluded by medical interview and a validated questionnaire for the Rome-II symptom criteria. The median age of the controls was 32 (range 20–48) years. Obesity was excluded in both patients and controls. Neither patients nor controls had been treated with antibiotics during one month prior to biopsy taking.

### Mucosa biopsy

Mucosa specimens from the proximal jejunum were taken in the time period from January 2006 to December 2009 with a sterile Watson capsule in all patients and controls. The Watson capsule was swallowed by the subject and brought by peristalsis to a position distal to the ligament of Treitz as determined by fluoroscopy. The Watson capsule was washed with sterile water before being opened. Biopsy samples obtained with capsules were divided into two pieces. One piece was frozen in liquid nitrogen and stored at −80°C for future DNA extraction. The other piece was fixed in formalin and mounted in paraffin blocks for histopathological analysis. The presence of villus atrophy and other significant abnormalities were excluded in all biopsies.

### DNA extraction

Extraction of total genomic DNA (gDNA) from frozen biopsies was performed with the DNeasy Blood & Tissue Kit (Qiagen, Germany). Biopsy samples were homogenized with a pestle in 1.5 ml tubes containing 200 µl freezing buffer. 100 µl of the homogenate was added to 200 µl lyses buffer (180 µl ATL buffer, 20 µl Proteinase K) and incubated overnight at 56°C in a shaking incubator. A negative extraction control was included for each batch of DNA extraction. After extraction of gDNA following the manufacturer's instruction the gDNA was eluted in 100 µl Buffer AE and stored at −20°C until further usage.

### PCR and template preparation for 454 sequencing

The protocol for barcoded 454-pyrosequencing has been adapted from Andersson et al.^21^ To create barcoded sequencing templates, PCR was performed using the forward primer 341f (GCCTTGCCAGCCCGCTCAGCCTACGGGNGGCWGCAG) and a barcoded reverse primer 805r (GCCTCCCTCGCGCCATCAGACTACHVGGGTATCTAATCC) targeting the V4 region of the 16S rRNA gene. For each sample, a PCR mix was prepared (triplicate) containing 1x PCR buffer, 1 mM dNTP's (Finnzymes, Finland), 1 U Phusion High-Fidelity DNA Polymerase (Finnzymes, Finland), 0.4 µM of each primer and 1–5 µl template DNA. For the PCR reaction the following conditions were applied: after an initial denaturation step (95°C, 5 min), 30 cycles of 95°C for 40 sec, 55°C for 40 sec and 72°C for 60 sec were followed by an final elongation step with 72°C for 10 min. Following agarose gel electrophoresis (1% agarose in TBE buffer containing gel red (Sigma Aldrich, Sweden), PCR products with the right size (approx. 500 bp) were excised and purified using the QIAquick gel extraction kit (Qiagen, Germany). The DNA concentration was assessed using the Qubit Fluorometer (Invitrogen, USA). After an initial concentration determination, the PCR products were diluted to a concentration between 3–5 ng/µl and then pooled for the 454-sequencing run. The final concentration of the DNA pool was measured for further dilutions for the emulsion PCR (emPCR) in the 454 workflow. Pyrosequencing was performed according to the Genome Sequencer FLX System Methods Manual.

### Bioinformatics pipeline

Pyrosequenced amplicon reads were de-multiplexed by stripping barcode and primer from each read. Reads which had one or more mismatches in barcode or primer were discarded. The remaining reads were quality filtered using the fastq_filter command of USEARCH v7.0.1001^45^ with parameters set to truncate reads to 200 basepairs whilst discarding shorter reads, and with maximum expected errors set to 0.5. The script pick_open_reference_otus.py from QIIME v1.7.0 was used to pick operational taxonomic units (OTUs)^46^. For this step the Greengenes May 2013 reference OTUs database at 97% identity was employed^47^. Chimera filtering was performed on the representative OTU reads using the uchime_ref command of USEARCH against the ChimeraSlayer “Gold” database from the Broad Microbiome Utilities r20110519^48^. The resulting data was analysed using R [http://www.R-project.org/] and the packages phyloseq 1.4.5, Metastats^23^, cluster 1.14.4, fpc 2.1-7, ggplot2 0.9.3.1, plyr 1.8.1, grid 3.0.1 and VGAM 0.9-3. Analyses at phylum and genus level were performed by: (1) aggregating all OTUs which had identical classification at the given taxonomic level (suggested classifications were heeded, e.g. [Prevotella] was considered identical with *Prevotella*), (2) normalizing samples by their total abundance, (3) discarding unclassified taxa and taxa with mean relative abundance lower than 0.01%. Step (3) was also performed on OTUs before analysis. Low abundance taxa were discarded to avoid having sampling depth effects influencing the analysis. Also, since the sample size was fairly small the analysis focused on taxa that were expressed in most samples in order to get meaningful results.

### Ethical considerations

The Regional Board of Research Ethics in Stockholm approved all parts of the study. Informed consent was obtained from all patients and controls and all methods were carried out in accordance with approved guidelines.

## Author Contributions

A.D. designed the study, collected the material, analyzed the data and wrote the paper, B.W. performed statistical analyses, analyzed the data and wrote the paper, E.L. performed the massive parallel sequencing, analyzed the data and revised the paper, K.Z. extracted DNA and barcoded samples and revised the paper, G.S. designed the study, provided the laboratory resources and revised the paper, W.Y. analyzed the data and revised the paper, L.E. designed the study, provided the laboratory resources and revised the paper, G.L. designed the study, obtained funding, collected the material, analyzed the data and wrote the paper.

## Supplementary Material

Supplementary Information Supplementary Figures

## Figures and Tables

**Table 1:** OTUs showing a trend towards differential expression between patients with IBS and controls. SEM = standard error of the mean

		Abundance (%) mean ± SEM		
OTU	Taxonomy	Patients	Controls	p (unadjusted)
4391262	Proteobacteria, *Escherichia* (genus)	2.78 ± 1.73	10.29 ± 2.56	0.080
4465561	Bacteroidetes, *Prevotella* (genus)	3.16 ± 0.51	1.56 ± 0.76	0.025
4425214	Firmicutes, *Streptococcus* (genus)	0.94 ± 0.29	2.27 ± 0.42	0.054
271159	Firmicutes, *Carnobacteriaceae* (family)	1.54 ± 0.41	0.73 ± 0.60	0.081
31235	Fusobacteria, *Leptotrichia* (genus)	1.14 ± 0.78	0.11 ± 1.15	0.077
