# Supplementary material for: No difference in small bowel microbiota between patients with irritable bowel syndrome and healthy controls
Source: Sci Rep. 2015 Feb 17;5:8508. doi: 10.1038/srep08508 (PMC4330528; doi:10.1038/srep08508)

Supplementary Figures

No difference in small bowel microbiota between patients with irritable bowel syndrome and healthy controls

Dlugosz A, Winckler B, Lundin E, Zakikhany K, Sandström G, Ye W, Engstrand L, Lindberg G.

Supplementary Figure 1. Cluster analysis of samples from IBS patients and controls using partitioning around medoids (PAM). A: PAM clustering of samples using Bray-Curtis distance. B: PAM clustering of samples using Jensen-Shannon divergence. C: PAM clustering of samples using weighted UniFrac distance.

Supplementary Figure 2: Multidimensional scaling (MDS). A: MDS coordinate 2 for Bray-Curtis distance indicates a separation of controls and patients, and coordinate 5 indicates a separation of controls from the IBS phenotype. B: MDS coordinate 2 for Jensen-Shannon divergence indicate a separation of controls and patients. C: MDS coordinate 2 for UniFrac indicate a separation of controls and patients

Supplementary Figure 1A

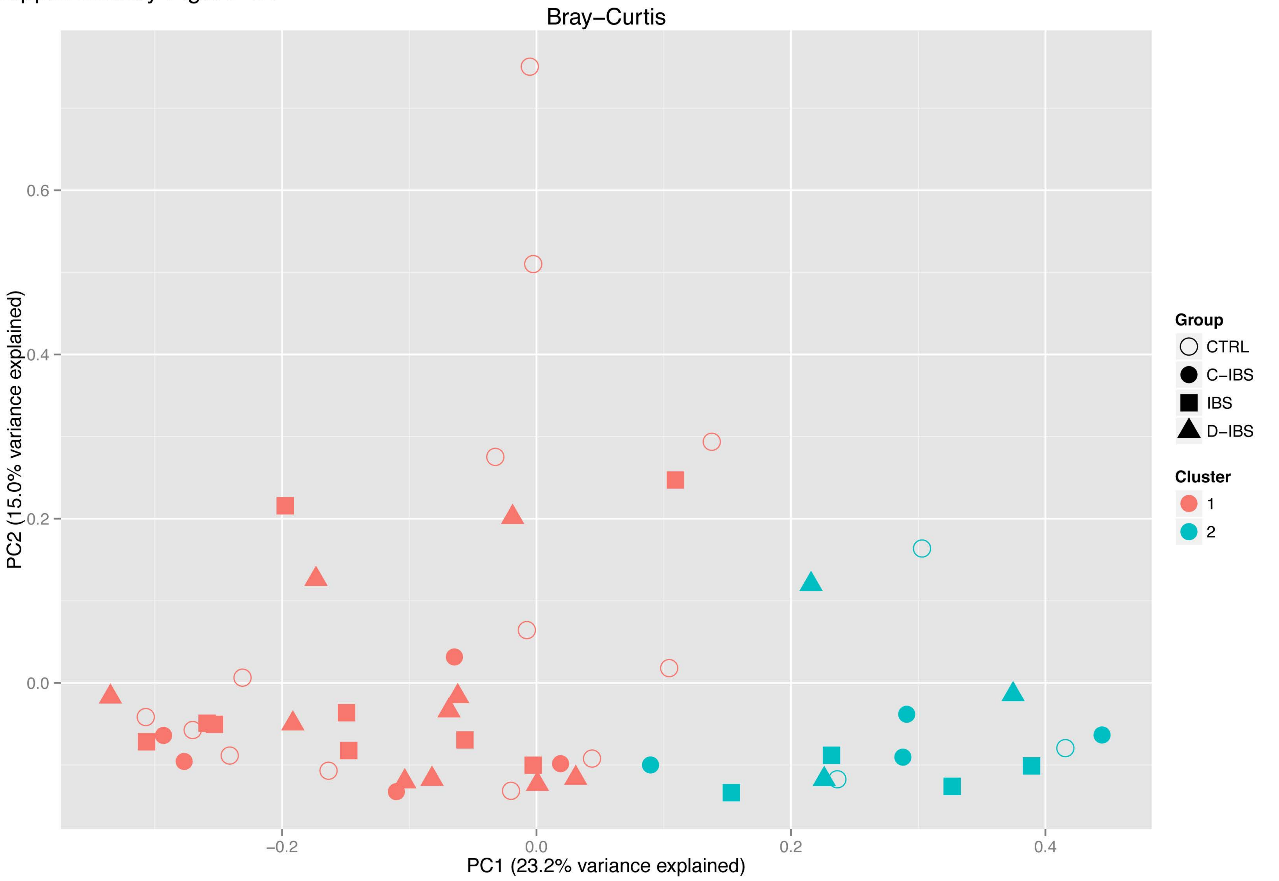

Supplementary Figure 1B

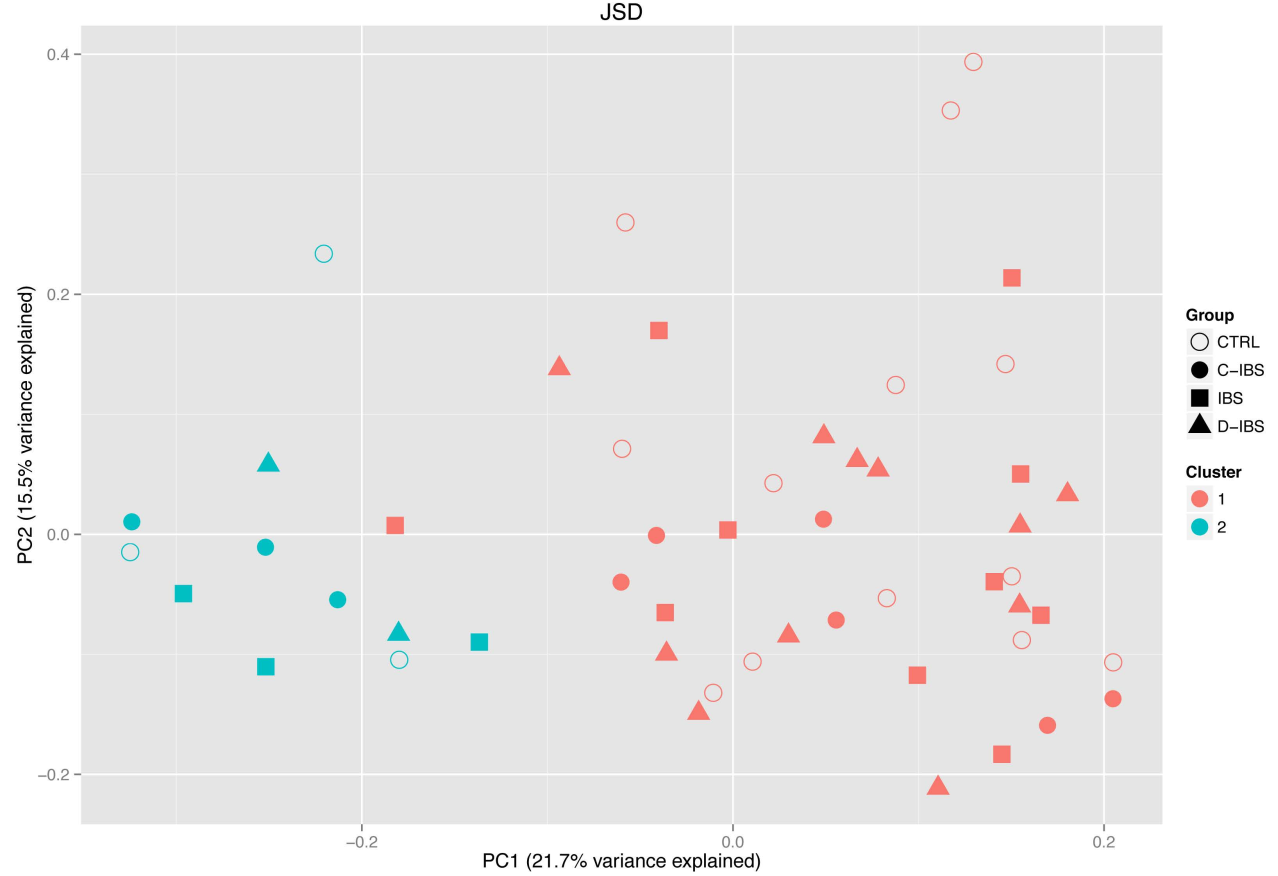

Supplementary Figure 1C

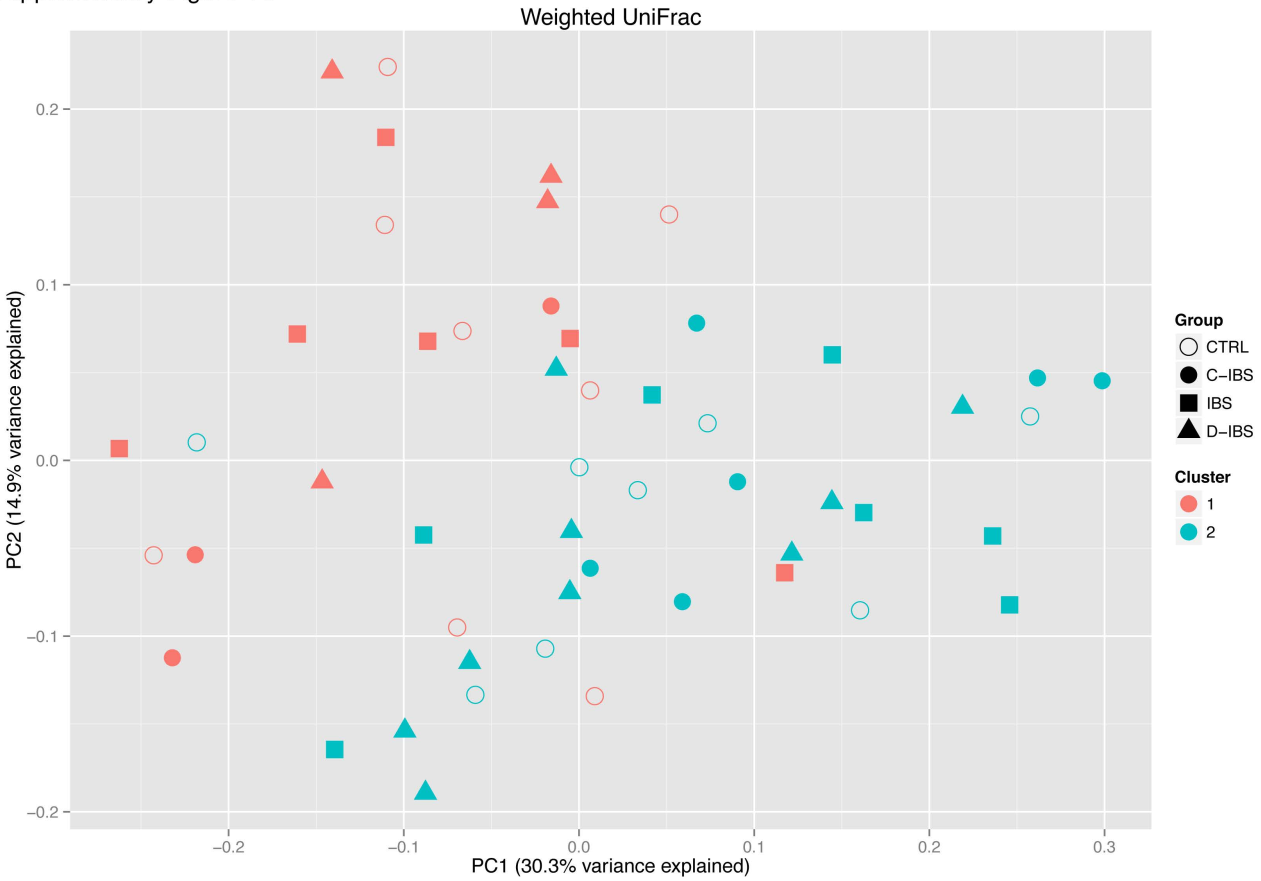

Supplementary Figure 2A

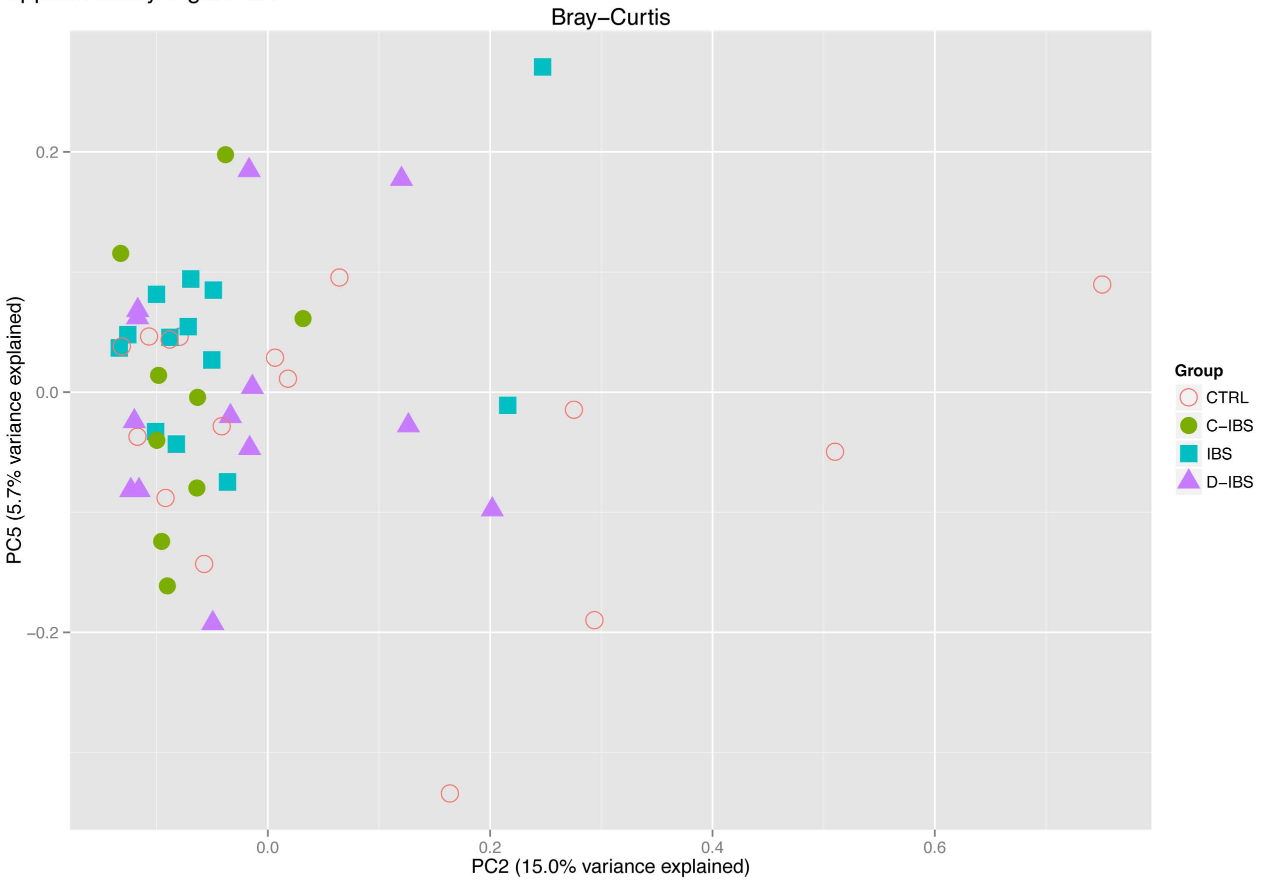

Supplementary Figure 2B

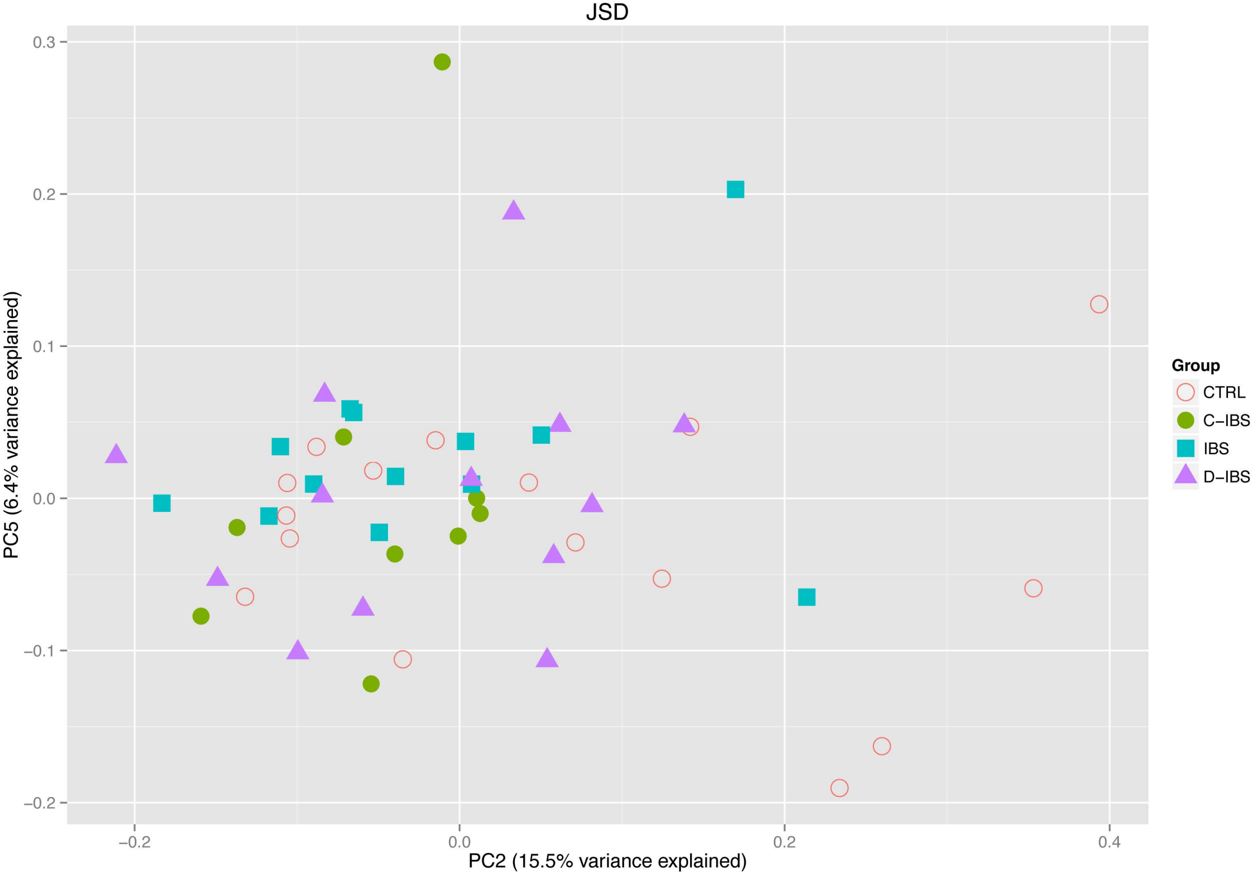

Supplementary Figure 2C

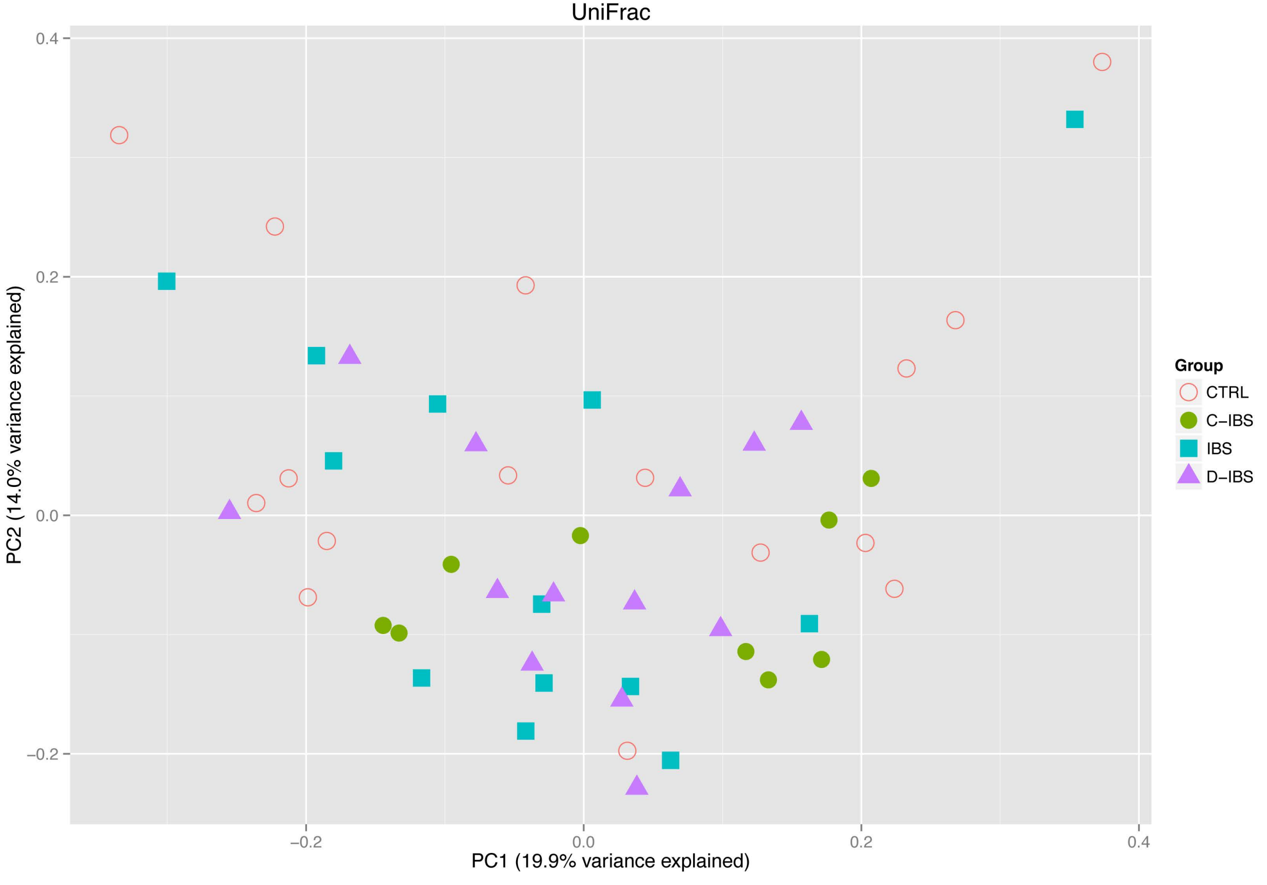

Supplement: Supplementary Information — Supplementary Figures [file srep08508-s1.pdf]
